# Supplementary material for: Global patterns and drivers of soil microbial nitrogen and phosphorus use efficiency
Source: Nat Commun. 2026 Mar 17;17:2576. doi: 10.1038/s41467-026-70602-0 (PMC12999993; doi:10.1038/s41467-026-70602-0)
Supplement: Supplementary file 1 — Supplementary Information [file 41467_2026_70602_MOESM1_ESM.pdf]

Supplementary Information

Global patterns and drivers of soil microbial nitrogen and phosphorus use efficiency

Decai Gao [1], Yakov Kuzyakov [2, 3], Manuel Delgado-Baquerizo [4], Josep Peñuelas [5, 6], Daryl L. Moorhead [7], Robert L. Sinsabaugh [8], Xiaofeng Xu [9], Lifei Sun [10], Huimin Wang [1, 11], Liang Kou [1, 11], Xiaoli Fu [1, 11], Xiaoqing Dai [1, 11], Shengwang Meng [1, 11], Ziping Liu [12], Siyu Wang [13], Frank Hagedorn [14], Matthias C. Rillig [15, 16], Yongxing Cui [15, *]

[1] Qianyanzhou Ecological Research Station, Key Laboratory of Ecosystem Network Observation and Modeling, Institute of Geographic Sciences and Natural Resources Research, Chinese Academy of Sciences, Beijing 100101, China

[2] Department of Soil Science of Temperate Ecosystems, Department of Agricultural Soil Science, University of Goettingen, Göttingen 37077, Germany

[3] Peoples Friendship University of Russia (RUDN University), Moscow 117198, Russia

[4] Laboratorio de Biodiversidad y Funcionamiento Ecosistémico. Instituto de Recursos Naturales y Agrobiología de Sevilla (IRNAS), Consejo Superior de Investigaciones Científicas (CSIC), Av. Reina Mercedes 10, Sevilla, Spain

[5] CSIC, Global Ecology Unit, CREAF-CSIC-UAB, Bellaterra (Catalonia) 08193, Spain

[6] CREAF, Cerdanyola del Valle`s (Catalonia) 08193, Spain

[7] Department of Environmental Sciences, University of Toledo, Toledo 43606, USA

[8] Biology Department, University of New Mexico, Albuquerque 87131, USA

[9] Biology Department, San Diego State University, San Diego, CA 92182, USA

[10] Key Laboratory of Environment Change and Resources Use in Beibu Gulf, Ministry of Education, and Guangxi Key Laboratory of Earth Surface Processes

and Intelligent Simulation of Nanning Normal University, Nanning 530001, China

[11] College of Resources and Environment, University of Chinese Academy of Sciences, Beijing 100190, China

[12] Key Laboratory of Geographical Processes and Ecological Security in Changbai Mountains, Ministry of Education, School of Geographical Sciences, Northeast Normal University, Changchun 130024, China

[13] School of Soil and Water Conservation, Beijing Forestry University, Beijing 100083, China

[14] Soil Biogeochemistry, Swiss Federal Institute for Forest, Snow and Landscape Research WSL, Birmensdorf 8903, Switzerland

[15] Institute of Biology, Freie Universität Berlin, Berlin 14195, Germany

[16] Berlin-Brandenburg Institute of Advanced Biodiversity Research (BBIB), Berlin, 14195, Germany

*** Corresponding author:**

Yongxing Cui

E-mail: cuiyongxing@zedat.fu-berlin.de

Institute of Biology, Freie Universität Berlin, Berlin 14195, Germany

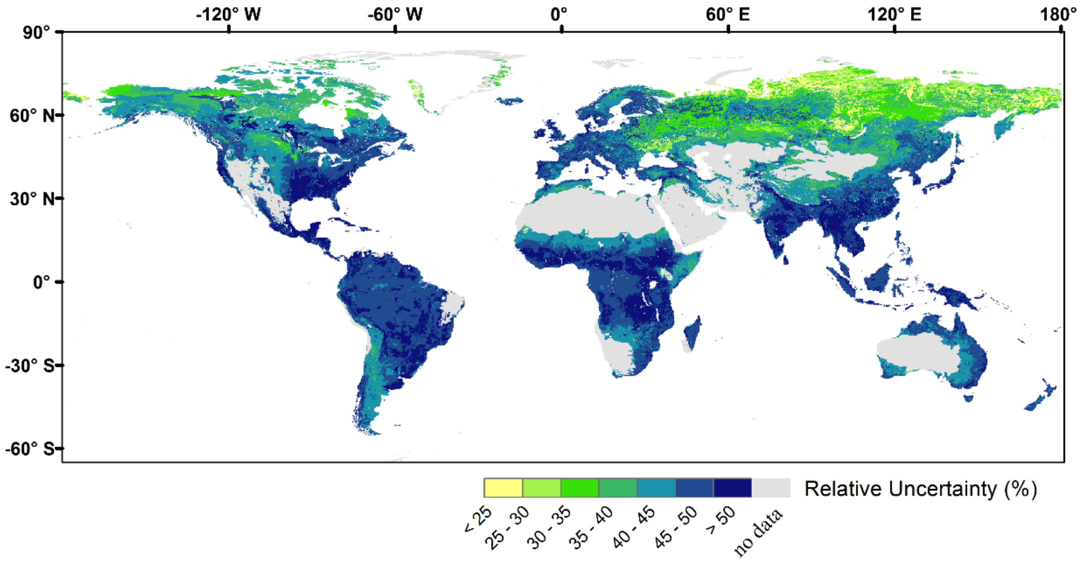

Supplementary Fig. 1 Relative uncertainty of predicted microbial nitrogen use efficiency (NUE). This map was predicted by a random forest model at a spatial resolution of 1 km × 1 km latitude–longitude. Relative uncertainty for each grid cell is calculated as the standard deviation (SD) of predictions across all 500 decision trees divided by the global mean NUE, expressed as percentage of global average.

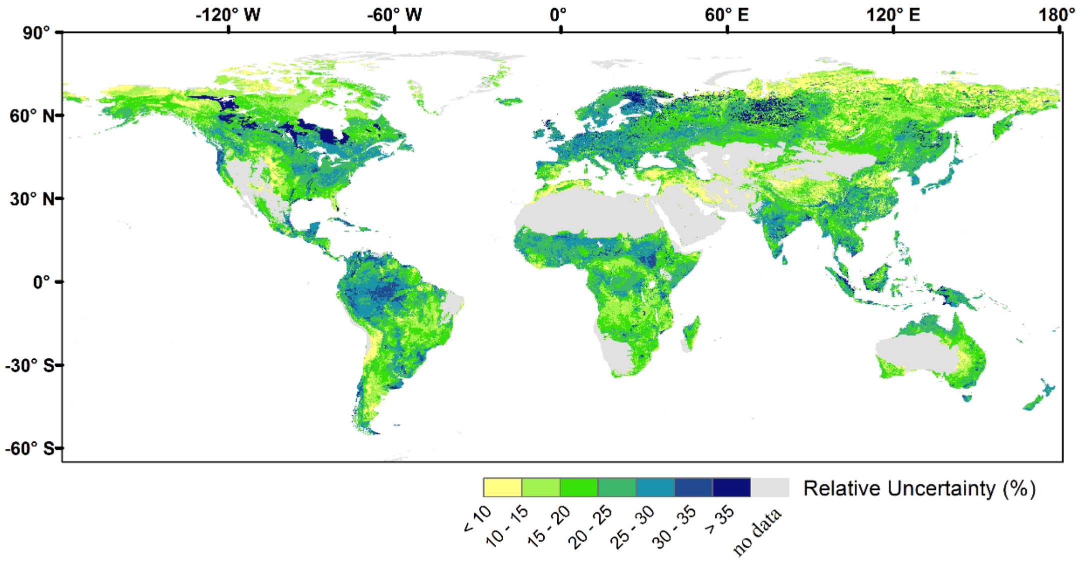

Supplementary Fig. 2 Relative uncertainty of predicted microbial phosphorus use efficiency (PUE). This map was predicted by a random forest model at a spatial resolution of 1 km × 1 km latitude–longitude. Relative uncertainty for each grid cell is calculated as the standard deviation (SD) of predictions across all 500 decision trees divided by the global mean PUE, expressed as percentage of global average.

Supplementary Table 1 Comparison of model performance among different models on microbial nitrogen use efficiency (NUE) and microbial phosphorus use efficiency (PUE). Four linear regression models (i.e., linear regression model, multiple stepwise regression model, least angle regression model, and elastic net model) and four nonlinear models (i.e., cubist model, boosted tree model, bagged tree model, and random forest model) were applied. R^2 and root mean square error (RMSE) were used to evaluate the performance of the models.

**Model**	**Microbial NUE**		**Microbial PUE**	
	R^2	RMSE	R^2	RMSE
Linear regression	0.18	0.18	0.37	0.22
Multiple stepwise regression	0.13	0.19	0.35	0.22
Least angle regression	0.18	0.18	0.37	0.22
Elastic net model	0.18	0.18	0.37	0.22
Cubist	0.74	0.11	0.82	0.12
Boosted tree	0.70	0.11	0.77	0.14
Bagged tree	0.57	0.13	0.59	0.18
Random forest	0.75	0.10	0.83	0.12

Supplementary Table 2 Gridded data used in this study. The environmental predictors include climate (mean annual temperature, mean annual precipitation, potential evapotranspiration, average annual relative humidity), vegetation (normalized difference vegetation index), soil microbial biomass (microbial biomass carbon, microbial biomass nitrogen, microbial biomass phosphorus), and soil properties (soil organic carbon, soil total nitrogen, soil total phosphorus, soil pH, soil clay, soil silt, cation exchange capacity, base saturation). All datasets were harmonized to a consistent spatial resolution of 1 km × 1 km using bilinear resampling for continuous variables and majority resampling for categorical data.

Type	Variable	Unit	Original resolution	Data Source
Climate	Mean annual temperature	℃	1 km^2	Ref[1]
	Mean annual precipitation	mm	1 km^2	Ref[1]
	Potential evapotranspiration	mm	1 km^2	Ref[2]
	Average annual relative humidity	%	0.08°	Ref[3]
Vegetation	Normalized difference vegetation index	-	0.05°	Ref[4]
Microbial biomass	Microbial biomass carbon, nitrogen, phosphorus	mg kg^{-1}	1 km^2	Ref[5]
Soil properties	Soil organic carbon	mg g^{-1}	250 m	Ref[6]
	Soil total nitrogen	mg g^{-1}	250 m	Ref[6]
	Soil total phosphorus	mg kg^{-1}	10 km	Ref[7]
	Soil pH	-	250 m	Ref[6]
	Soil silt	%	250 m	Ref[6]
	Soil clay	%	250 m	Ref[6]
	Cation exchange capacity	cmol kg^{-1}	250 m	Ref[6]
	Base saturation	%	250 m	Ref[6]

Supplementary Table 3 *p*-values for the relative importance of climatic, vegetation, and soil factors for microbial nitrogen use efficiency (NUE) and phosphorus use efficiency (PUE). Climatic factors include: MAP, mean annual precipitation (mm); MAT, mean annual temperature (℃); PET, potential evapotranspiration (mm); humidity, average annual relative humidity (%). Soil factors include: SOC, soil organic carbon (%);pH, soil pH; silt, soil silt content (%); clay, soil clay content (%); CEC, cation exchange capacity (cmol kg^{-1}); Bsat, base saturation (%); depth, soil depth (cm).Vegetation factor includes: NDVI, normalized difference vegetation index.%IncMSE, percent increase in mean squared error (i.e., mean decrease in accuracy). Statistical significance was assessed using one-sided permutation tests (500 permutations), with *p*-values representing the probability of obtaining an importance score greater than expected by chance under the null distribution.

Predictors	Microbial NUE		Microbial PUE	
	%IncMSE	*p*-value	%IncMSE	*p*-value
MAT	46.93	0.009901	60.80	0.009901
MAP	43.08	0.009901	50.62	0.009901
PET	47.31	0.009901	44.22	0.009901
Humidity	51.46	0.009901	50.82	0.009901
NDVI	50.52	0.009901	58.11	0.009901
pH	31.50	0.009901	26.73	0.009901
SOC	64.00	0.009901	65.48	0.029604
Clay	31.28	0.009901	39.74	0.009901
Silt	44.06	0.009901	39.86	0.009901
Bsat	23.43	0.039901	37.24	0.009901
CEC	31.53	0.009901	37.54	0.009901
Depth	45.24	0.009901	53.11	0.009901

Supplementary References:

1. Karger, D. et al. Climatologies at high resolution for the earth's land surface areas. *Sci. Data* **4**, 170122 (2017). https://doi.org/10.1038/sdata.2017.122
2. Zomer, R.J., Xu, J. & Trabucco, A. Version 3 of the Global Aridity Index and Potential Evapotranspiration Database. *Sci. Data* **9**, 409 (2022). https://doi.org/10.1038/s41597-022-01493-1
3. New, M., Lister, D., Hulme, M. & Makin, I., 2002. A high-resolution data set of surface climate over global land areas. *Clim. Res.* **21**, 1–25 (2002). https://doi.org/10.3354/cr021001
4. Didan, K. & Barreto, A. NASA MEaSUREs Vegetation Index and Phenology (VIP) Vegetation Indices Monthly Global 0.05Deg CMG [Data set] (NASA EOSDIS Land Process DAAC, 2019). https://doi.org/10.5067/MEaSUREs/VIP/VIP30.004
5. Gao, D. et al. Three-dimensional mapping of carbon, nitrogen, and phosphorus in soil microbial biomass and their stoichiometry at the global scale. *Glob. Change Biol.* **28**, 6728-6740 (2022). https://doi.org/10.1111/gcb.16374
6. Hengl, T. et al. SoilGrids250m: Global gridded soil information based on machine learning. *PLoS One*, **12**, e0169748 (2017). https://doi.org/10.1371/journal.pone.0169748
7. Miralles, D. G. *et al.* GLEAM4: global land evaporation and soil moisture dataset at 0.1° resolution from 1980 to near present. *Sci. Data* **12**, 416 (2025).
