# Supplementary material for: Global patterns and drivers of soil microbial nitrogen and phosphorus use efficiency
Source: Nat Commun. 2026 Mar 17;17:2576. doi: 10.1038/s41467-026-70602-0 (PMC12999993; doi:10.1038/s41467-026-70602-0)
Supplement: Supplementary file 2 — Description of Additional Supplementary Information [file 41467_2026_70602_MOESM2_ESM.pdf]

Description of Additional Supplementary Information

Title: Supplementary Data 1

Description: Comprehensive list of primary studies included in this analysis. The file contains bibliographic information for all original publications (*n* = 212) from which data were extracted, including authors, year, journal, title, and DOI. These references constitute the complete dataset underlying the analyses presented in this study.
