# Supplementary material for: Global patterns and drivers of soil microbial nitrogen and phosphorus use efficiency
Source: Nat Commun. 2026 Mar 17;17:2576. doi: 10.1038/s41467-026-70602-0 (PMC12999993; doi:10.1038/s41467-026-70602-0)
Supplement: Supplementary file 3 — Supplementary Data 1 [file 41467_2026_70602_MOESM3_ESM.docx]

Acosta-Martínez, V., Cruz, L., Sotomayor-Ramírez, D., & Pérez-Alegría, L. (2007). Enzyme activities as affected by soil properties and land use in a tropical watershed. *Applied Soil Ecology, 35*(1), 35-45. doi:10.1016/j.apsoil.2006.05.012

Adamczyk, B., Kilpeläinen, P., Kitunen, V., & Smolander, A. (2014). Potential activities of enzymes involved in N, C, P and S cycling in boreal forest soil under different tree species. *Pedobiologia, 57*(2), 97-102. doi:10.1016/j.pedobi.2013.12.003

Asensio, D., Zuccarini, P., Ogaya, R., Marañón-Jiménez, S., Sardans, J., & Peñuelas, J. (2021). Simulated climate change and seasonal drought increase carbon and phosphorus demand in Mediterranean forest soils. *Soil Biology and Biochemistry, 163*. doi:10.1016/j.soilbio.2021.108424

Banerjee, S., Bora, S., Thrall, P. H., & Richardson, A. E. (2016). Soil C and N as causal factors of spatial variation in extracellular enzyme activity across grassland-woodland ecotones. *Applied Soil Ecology, 105*, 1-8. doi:10.1016/j.apsoil.2016.04.002

Bell, C., Carrillo, Y., Boot, C. M., Rocca, J. D., Pendall, E., & Wallenstein, M. D. (2014). Rhizosphere stoichiometry: are C : N : P ratios of plants, soils, and enzymes conserved at the plant species-level? *New Phytologist, 201*(2), 505-517. doi:10.1111/nph.12531

Bell, T. H., & Henry, H. A. L. (2011). Fine scale variability in soil extracellular enzyme activity is insensitive to rain events and temperature in a mesic system. *Pedobiologia, 54*(2), 141-146. doi:10.1016/j.pedobi.2010.12.003

Bell, T. H., Klironomos, J. N., & Henry, H. A. L. (2010). Seasonal Responses of Extracellular Enzyme Activity and Microbial Biomass to Warming and Nitrogen Addition. *Soil Science Society of America Journal, 74*(3), 820-828. doi:10.2136/sssaj2009.0036

Benvenutto‐Vargas, V. P., Ochoa‐Hueso, R., & Power, S. (2020). Effects of nitrogen deposition on the spatial pattern of biocrusts and soil microbial activity in a semi‐arid Mediterranean shrubland. *Functional Ecology, 34*(4), 923-937. doi:10.1111/1365-2435.13512

Boeddinghaus, R. S., Nunan, N., Berner, D., Marhan, S., & Kandeler, E. (2015). Do general spatial relationships for microbial biomass and soil enzyme activities exist in temperate grassland soils? *Soil Biology and Biochemistry, 88*, 430-440. doi:10.1016/j.soilbio.2015.05.026

Boot, C. M., Hall, E. K., Denef, K., & Baron, J. S. (2016). Long-term reactive nitrogen loading alters soil carbon and microbial community properties in a subalpine forest ecosystem. *Soil Biology and Biochemistry, 92*, 211-220. doi:10.1016/j.soilbio.2015.10.002

Bowles, T. M., Acosta-Martínez, V., Calderón, F., & Jackson, L. E. (2014). Soil enzyme activities, microbial communities, and carbon and nitrogen availability in organic agroecosystems across an intensively-managed agricultural landscape. *Soil Biology and Biochemistry, 68*, 252-262. doi:10.1016/j.soilbio.2013.10.004

Bragazza, L., Parisod, J., Buttler, A., & Bardgett, R. D. (2012). Biogeochemical plant–soil microbe feedback in response to climate warming in peatlands. *Nature Climate Change, 3*(3), 273-277. doi:10.1038/nclimate1781

Broadbent, A. A. D., Snell, H. S. K., Michas, A., Pritchard, W. J., Newbold, L., Cordero, I., . . . Bardgett, R. D. (2021). Climate change alters temporal dynamics of alpine soil microbial functioning and biogeochemical cycling via earlier snowmelt. *The* *ISME Journal, 15*(8), 2264-2275. doi:10.1038/s41396-021-00922-0

Burke, D. J., Smemo, K. A., López-Gutiérrez, J. C., & DeForest, J. L. (2012). Soil fungi influence the distribution of microbial functional groups that mediate forest greenhouse gas emissions. *Soil Biology and Biochemistry, 53*, 112-119. doi:10.1016/j.soilbio.2012.05.008

Buscardo, E., Souza, R. C., Meir, P., Geml, J., Schmidt, S. K., da Costa, A. C. L., & Nagy, L. (2021). Effects of natural and experimental drought on soil fungi and biogeochemistry in an Amazon rain forest. *Communications Earth & Environment, 2*(1). doi:10.1038/s43247-021-00124-8

Cao, Z., Jia, Y., Cai, Y., Wang, X., Hu, H., Zhang, J., . . . Feng, X. (2019). Past aridity's effect on carbon mineralization potentials in grassland soils. *Biogeosciences, 16*(18), 3605-3619. doi:10.5194/bg-16-3605-2019

Čapek, P., Diáková, K., Dickopp, J.-E., Bárta, J., Wild, B., Schnecker, J., . . . Šantrůčková, H. (2015). The effect of warming on the vulnerability of subducted organic carbon in arctic soils. *Soil Biology and Biochemistry, 90*, 19-29. doi:10.1016/j.soilbio.2015.07.013

Carnovale, D., Richardson, A. E., Thrall, P. H., Bissett, A., & Baker, G. (2021). Litter decomposition and microbial communities alters depending on litter type and overstory species in revegetated agricultural land. *Pedobiologia, 84*. doi:10.1016/j.pedobi.2020.150702

Carrara, J. E., Walter, C. A., Freedman, Z. B., Hostetler, A. N., Hawkins, J. S., Fernandez, I. J., & Brzostek, E. R. (2021). Differences in microbial community response to nitrogen fertilization result in unique enzyme shifts between arbuscular and ectomycorrhizal-dominated soils. *Global Change Biology, 27*(10), 2049-2060. doi:10.1111/gcb.15523

Carrara, J. E., Walter, C. A., Hawkins, J. S., Peterjohn, W. T., Averill, C., & Brzostek, E. R. (2018). Interactions among plants, bacteria, and fungi reduce extracellular enzyme activities under long-term N fertilization. *Global Change Biology, 24*(6), 2721-2734. doi:10.1111/gcb.14081

Carrillo, Y., Bell, C., Koyama, A., Canarini, A., Boot, C. M., Wallenstein, M., . . . de Vries, F. (2017). Plant traits, stoichiometry and microbes as drivers of decomposition in the rhizosphere in a temperate grassland. *Journal of Ecology, 105*(6), 1750-1765. doi:10.1111/1365-2745.12772

Cenini, V. L., Fornara, D. A., McMullan, G., Ternan, N., Lajtha, K., & Crawley, M. J. (2015). Chronic nitrogen fertilization and carbon sequestration in grassland soils: evidence of a microbial enzyme link. *Biogeochemistry, 126*(3), 301-313. doi:10.1007/s10533-015-0157-5

Chen, H., Li, D., Mao, Q., Xiao, K., & Wang, K. (2019). Resource limitation of soil microbes in karst ecosystems. *Science of the Total Environment, 650*(Pt 1), 241-248. doi:10.1016/j.scitotenv.2018.09.036

Chen, H., Li, D., Xiao, K., Wang, K., & Treseder, K. (2018). Soil microbial processes and resource limitation in karst and non‐karst forests. *Functional Ecology, 32*(5), 1400-1409. doi:10.1111/1365-2435.13069

Chen, H., Zheng, M., Mao, Q., Xiao, K., Wang, K., & Li, D. (2019). Cropland conversion changes the status of microbial resource limitation in degraded karst soil. *Geoderma, 352*, 197-203. doi:10.1016/j.geoderma.2019.06.018

Chen, J., Xiao, W., Zheng, C., & Zhu, B. (2020). Nitrogen addition has contrasting effects on particulate and mineral-associated soil organic carbon in a subtropical forest. *Soil Biology and Biochemistry, 142*. doi:10.1016/j.soilbio.2020.107708

Chen, W., Zhou, H., Wu, Y., Wang, J., Zhao, Z., Li, Y., . . . Xue, S. (2020). Direct and indirect influences of long-term fertilization on microbial carbon and nitrogen cycles in an alpine grassland. *Soil Biology and Biochemistry, 149*. doi:10.1016/j.soilbio.2020.107922

Chen, X., Ding, Z., Tang, M., & Zhu, B. (2018). Greater variations of rhizosphere effects within mycorrhizal group than between mycorrhizal group in a temperate forest. *Soil Biology and Biochemistry, 126*, 237-246. doi:10.1016/j.soilbio.2018.08.026

Chen, X., Han, X., Lu, X., Yan, J., Biswas, A., & Zou, W. (2021). Long-term continuous cropping affects ecoenzymatic stoichiometry of microbial nutrient acquisition: a case study from a Chinese Mollisol. *Journal of the Science of Food and Agriculture, 101*(15), 6338-6346. doi:10.1002/jsfa.11304

Chernysheva, E., Khomutova, T., Fornasier, F., Kuznetsova, T., & Borisov, A. (2018). Effects of long-term medieval agriculture on soil properties: A case study from the Kislovodsk basin, Northern Caucasus, Russia. *Journal of Mountain Science, 15*(6), 1171-1185. doi:10.1007/s11629-017-4666-7

Chung, H., Zak, D. R., & Lilleskov, E. A. (2006). Fungal community composition and metabolism under elevated CO_2_ and O_3_. *Oecologia, 147*(1), 143-154. doi:10.1007/s00442-005-0249-3

Cui, Y., Bing, H., Fang, L., Jiang, M., Shen, G., Yu, J., . . . Zhang, X. (2019). Extracellular enzyme stoichiometry reveals the carbon and phosphorus limitations of microbial metabolisms in the rhizosphere and bulk soils in alpine ecosystems. *Plant and Soil, 458*(1-2), 7-20. doi:10.1007/s11104-019-04159-x

Cui, Y., Bing, H., Moorhead, D. L., Delgado-Baquerizo, M., Ye, L., Yu, J., . . . Fang, L. (2022). Ecoenzymatic stoichiometry reveals widespread soil phosphorus limitation to microbial metabolism across Chinese forests. *Communications Earth & Environment, 3*(1). doi:10.1038/s43247-022-00523-5

Cui, Y., Fang, L., Deng, L., Guo, X., Han, F., Ju, W., . . . Zhang, X. (2019). Patterns of soil microbial nutrient limitations and their roles in the variation of soil organic carbon across a precipitation gradient in an arid and semi-arid region. *Science of the Total Environment, 658*, 1440-1451. doi:10.1016/j.scitotenv.2018.12.289

Cui, Y., Fang, L., Guo, X., Han, F., Ju, W., Ye, L., . . . Zhang, X. (2019). Natural grassland as the optimal pattern of vegetation restoration in arid and semi-arid regions: Evidence from nutrient limitation of soil microbes. *Science of the Total Environment, 648*, 388-397. doi:10.1016/j.scitotenv.2018.08.173

Cui, Y., Fang, L., Guo, X., Wang, X., Wang, Y., Li, P., . . . Zhang, X. (2018). Responses of soil microbial communities to nutrient limitation in the desert-grassland ecological transition zone. *Science of the Total Environment, 642*, 45-55. doi:10.1016/j.scitotenv.2018.06.033

Cui, Y., Wang, X., Zhang, X., Ju, W., Duan, C., Guo, X., . . . Fang, L. (2020). Soil moisture mediates microbial carbon and phosphorus metabolism during vegetation succession in a semiarid region. *Soil Biology and Biochemistry, 147*. doi:10.1016/j.soilbio.2020.107814

Cui, Y., Zhang, Y., Duan, C., Wang, X., Zhang, X., Ju, W., . . . Fang, L. (2020). Ecoenzymatic stoichiometry reveals microbial phosphorus limitation decreases the nitrogen cycling potential of soils in semi-arid agricultural ecosystems. *Soil and Tillage Research, 197*. doi:10.1016/j.still.2019.104463

Cui, Y. X., Fang, L. C., Guo, X. B., Wang, X., Zhang, Y. J., Li, P. F., & Zhang, X. C. (2018). Ecoenzymatic stoichiometry and microbial nutrient limitation in rhizosphere soil in the arid area of the northern Loess Plateau, China. *Soil Biology & Biochemistry, 116*, 11-21. doi:10.1016/j.soilbio.2017.09.025

Cusack, D. F., Silver, W. L., Torn, M. S., Burton, S. D., & Firestone, M. K. (2011). Changes in microbial community characteristics and soil organic matter with nitrogen additions in two tropical forests. *Ecology, 92*(3), 621-632. doi:10.1890/10-0459.1

Cusack, D. F., Torn, M. S., McDowell, W. H., & Silver, W. L. (2010). The response of heterotrophic activity and carbon cycling to nitrogen additions and warming in two tropical soils. *Global Change Biology, 16*(9), 2555-2572. doi:10.1111/j.1365-2486.2009.02131.x

Das Gupta, S., MacKenzie, M. D., & Quideau, S. A. (2015). Using spatial ecology to examine above and belowground interactions on a reclaimed aspen stand in northern Alberta. *Geoderma, 259-260*, 12-22. doi:10.1016/j.geoderma.2015.04.004

DeForest, J. L., & Moorhead, D. L. (2020). Effects of elevated pH and phosphorus fertilizer on soil C, N and P enzyme stoichiometry in an acidic mixed mesophytic deciduous forest. *Soil Biology and Biochemistry, 150*. doi:10.1016/j.soilbio.2020.107996

DeForest, J. L., Zak, D. R., Pregitzer, K. S., & Burton, A. J. (2004). Atmospheric Nitrate Deposition, Microbial Community Composition, and Enzyme Activity in Northern Hardwood Forests. *Soil Science Society of America Journal, 68*(1), 132-138. doi:10.2136/sssaj2004.1320

Delgado‐Baquerizo, M., Trivedi, P., Trivedi, C., Eldridge, D. J., Reich, P. B., Jeffries, T. C., . . . Bennett, A. (2017). Microbial richness and composition independently drive soil multifunctionality. *Functional Ecology, 31*(12), 2330-2343. doi:10.1111/1365-2435.12924

Deng, L., Peng, C., Huang, C., Wang, K., Liu, Q., Liu, Y., . . . Shangguan, Z. (2019). Drivers of soil microbial metabolic limitation changes along a vegetation restoration gradient on the Loess Plateau, China. *Geoderma, 353*, 188-200. doi:10.1016/j.geoderma.2019.06.037

Ding, L., Shang, Y., Zhang, W., Zhang, Y., Li, S., Wei, X., . . . Wang, P. (2020). Disentangling the effects of driving forces on soil bacterial and fungal communities under shrub encroachment on the Guizhou Plateau of China. *Science of the Total Environment, 709*, 136207. doi:10.1016/j.scitotenv.2019.136207

Dong, C. C., Wang, W., Liu, H. Y., Xu, X. T., & Zeng, H. (2019). Temperate grassland shifted from nitrogen to phosphorus limitation induced by degradation and nitrogen deposition: Evidence from soil extracellular enzyme stoichiometry. *Ecological Indicators, 101*, 453-464. doi:10.1016/j.ecolind.2019.01.046

Dorodnikov, M., Blagodatskaya, E., Blagodatsky, S., Marhan, S., Fangmeier, A., & Kuzyakov, Y. (2009). Stimulation of microbial extracellular enzyme activities by elevated CO_2_ depends on soil aggregate size. *Global Change Biology, 15*(6), 1603-1614. doi:10.1111/j.1365-2486.2009.01844.x

Dove, N. C., Arogyaswamy, K., Billings, S. A., Botthoff, J. K., Carey, C. J., Cisco, C., . . . Aronson, E. L. (2020). Continental-scale patterns of extracellular enzyme activity in the subsoil: an overlooked reservoir of microbial activity. *Environmental Research Letters, 15*(10). doi:10.1088/1748-9326/abb0b3

Dove, N. C., Stark, J. M., Newman, G. S., & Hart, S. C. (2019). Carbon control on terrestrial ecosystem function across contrasting site productivities: the carbon connection revisited. *Ecology, 100*(7), e02695. doi:10.1002/ecy.2695

Du, Z., Wang, W., Zeng, W., & Zeng, H. (2014). Nitrogen deposition enhances carbon sequestration by plantations in northern China. *PLoS One, 9*(2), e87975. doi:10.1371/journal.pone.0087975

Elzobair, K. A., Stromberger, M. E., Ippolito, J. A., & Lentz, R. D. (2016). Contrasting effects of biochar versus manure on soil microbial communities and enzyme activities in an Aridisol. *Chemosphere, 142*, 145-152. doi:10.1016/j.chemosphere.2015.06.044

Fatemi, F. R., Fernandez, I. J., Simon, K. S., & Dail, D. B. (2016). Nitrogen and phosphorus regulation of soil enzyme activities in acid forest soils. *Soil Biology and Biochemistry, 98*, 171-179. doi:10.1016/j.soilbio.2016.02.017

Feng, J., Wei, K., Chen, Z., Lü, X., Tian, J., Wang, C., & Chen, L. (2019). Coupling and Decoupling of Soil Carbon and Nutrient Cycles Across an Aridity Gradient in the Drylands of Northern China: Evidence From Ecoenzymatic Stoichiometry. *Global Biogeochemical Cycles, 33*(5), 559-569. doi:10.1029/2018gb006112

Feng, X., Zhang, L., Zhao, F., Bai, H., & Doughty, R. (2019). Effects of mixing feldspathic sandstone and sand on soil microbial biomass and extracellular enzyme activities—a case study in Mu Us sandy land in China. *Applied Sciences, 9*(19). doi:10.3390/app9193963

Fernández-García, V., Marcos, E., Huerta, S., & Calvo, L. (2021). Soil-vegetation relationships in Mediterranean forests after fire. *Forest Ecosystems, 8*(1). doi:10.1186/s40663-021-00295-y

Finzi, A. C., Sinsabaugh, R. L., Long, T. M., & Osgood, M. P. (2006). Microbial community responses to atmospheric carbon dioxide enrichment in a warm-temperate forest. *Ecosystems, 9*(2), 215-226. doi:10.1007/s10021-005-0078-6

Forstner, S. J., Wechselberger, V., Stecher, S., Muller, S., Keiblinger, K. M., Wanek, W., . . . Zechmeister-Boltenstern, S. (2019). Resistant soil microbial communities show signs of increasing phosphorus limitation in two temperate forests after long-term nitrogen addition. *Frontiers in Forests and Global Change, 2*. doi:10.3389/ffgc.2019.00073

Fujita, K., Miyabara, Y., & Kunito, T. (2019). Microbial biomass and ecoenzymatic stoichiometries vary in response to nutrient availability in an arable soil. *European Journal of Soil Biology, 91*, 1-8. doi:10.1016/j.ejsobi.2018.12.005

Grandy, A. S., Strickland, M. S., Lauber, C. L., Bradford, M. A., & Fierer, N. (2009). The influence of microbial communities, management, and soil texture on soil organic matter chemistry. *Geoderma, 150*(3-4), 278-286. doi:10.1016/j.geoderma.2009.02.007

Guan, H. L., Fan, J. W., & Lu, X. (2022). Soil specific enzyme stoichiometry reflects nitrogen limitation of microorganisms under different types of vegetation restoration in the karst areas. *Applied Soil Ecology, 169*. doi:10.1016/j.apsoil.2021.104253

Guan, P., Yang, J., Yang, Y., Wang, W., Zhang, P., & Wu, D. (2020). Land conversion from cropland to grassland alleviates climate warming effects on nutrient limitation: Evidence from soil enzymatic activity and stoichiometry. *Global Ecology and Conservation, 24*. doi:10.1016/j.gecco.2020.e01328

Guerrero-Ramírez, N. R., Pizarro, V., & Turner, B. L. (2020). Soil and microbial nutrient status are heterogeneous within an elevational belt on a neotropical mountain. *Pedobiologia, 83*. doi:10.1016/j.pedobi.2020.150689

Guo, J., McCulley, R. L., Phillips, T. D., & McNear, D. H. (2016). Fungal endophyte and tall fescue cultivar interact to differentially affect bulk and rhizosphere soil processes governing C and N cycling. *Soil Biology and Biochemistry, 101*, 165-174. doi:10.1016/j.soilbio.2016.07.014

Guo, K., Zhao, Y., Liu, Y., Chen, J., Wu, Q., Ruan, Y., . . . Qin, H. (2020). Pyrolysis temperature of biochar affects ecoenzymatic stoichiometry and microbial nutrient-use efficiency in a bamboo forest soil. *Geoderma, 363*. doi:10.1016/j.geoderma.2019.114162

Gutknecht, J. L. M., Henry, H. A. L., & Balser, T. C. (2010). Inter-annual variation in soil extra-cellular enzyme activity in response to simulated global change and fire disturbance. *Pedobiologia, 53*(5), 283-293. doi:10.1016/j.pedobi.2010.02.001

Hall, S. J., Ahmed, B., Ortiz, P., Davies, R., Sponseller, R. A., & Grimm, N. B. (2009). Urbanization alters soil microbial functioning in the sonoran desert. *Ecosystems, 12*(4), 654-671. doi:10.1007/s10021-009-9249-1

Hasegawa, S., Macdonald, C. A., & Power, S. A. (2016). Elevated carbon dioxide increases soil nitrogen and phosphorus availability in a phosphorus-limited Eucalyptus woodland. *Global Change Biology, 22*(4), 1628-1643. doi:10.1111/gcb.13147

Herold, N., Schöning, I., Berner, D., Haslwimmer, H., Kandeler, E., Michalzik, B., & Schrumpf, M. (2014). Vertical gradients of potential enzyme activities in soil profiles of European beech, Norway spruce and Scots pine dominated forest sites. *Pedobiologia, 57*(3), 181-189. doi:10.1016/j.pedobi.2014.03.003

Heuck, C., Smolka, G., Whalen, E. D., Frey, S., Gundersen, P., Moldan, F., . . . Spohn, M. (2018). Effects of long-term nitrogen addition on phosphorus cycling in organic soil horizons of temperate forests. *Biogeochemistry, 141*(2), 167-181. doi:10.1007/s10533-018-0511-5

Hewins, D. B., Fatemi, F., Adams, B., Carlyle, C. N., Chang, S. X., & Bork, E. W. (2015). Grazing, regional climate and soil biophysical impacts on microbial enzyme activity in grassland soil of western Canada. *Pedobiologia, 58*(5-6), 201-209. doi:10.1016/j.pedobi.2015.10.003

Hill, B. H., Elonen, C. M., Jicha, T. M., Kolka, R. K., Lehto, L. L. P., Sebestyen, S. D., & Seifert-Monson, L. R. (2014). Ecoenzymatic stoichiometry and microbial processing of organic matter in northern bogs and fens reveals a common P-limitation between peatland types. *Biogeochemistry, 120*(1-3), 203-224. doi:10.1007/s10533-014-9991-0

Hobbie, S. E., Eddy, W. C., Buyarski, C. R., Adair, E. C., Ogdahl, M. L., & Weisenhorn, P. (2012). Response of decomposing litter and its microbial community to multiple forms of nitrogen enrichment. *Ecological Monographs, 82*(3), 389-405. doi:10.1890/11-1600.1

Hsiao, C. J., Sassenrath, G. F., Zeglin, L. H., Hettiarachchi, G. M., & Rice, C. W. (2018). Vertical changes of soil microbial properties in claypan soils. *Soil Biology & Biochemistry, 121*, 154-164. doi:10.1016/j.soilbio.2018.03.012

Hu, Z., Li, J., Shi, K., Ren, G., Dai, Z., Sun, J., . . . Du, D. (2021). Effects of Canada goldenrod invasion on soil extracellular enzyme activities and ecoenzymatic stoichiometry. *Sustainability, 13*(7). doi:10.3390/su13073768

Ivashchenko, K., Sushko, S., Selezneva, A., Ananyeva, N., Zhuravleva, A., Kudeyarov, V., . . . Blagodatsky, S. (2021). Soil microbial activity along an altitudinal gradient: Vegetation as a main driver beyond topographic and edaphic factors. *Applied Soil Ecology, 168*. doi:10.1016/j.apsoil.2021.104197

Jian, S., Li, J., Chen, J., Wang, G., Mayes, M. A., Dzantor, K. E., . . . Luo, Y. (2016). Soil extracellular enzyme activities, soil carbon and nitrogen storage under nitrogen fertilization: A meta-analysis. *Soil Biology and Biochemistry, 101*, 32-43. doi:10.1016/j.soilbio.2016.07.003

Jian, Z., Ni, Y., Zeng, L., Lei, L., Xu, J., Xiao, W., & Li, M.-H. (2021). Latitudinal patterns of soil extracellular enzyme activities and their controlling factors in Pinus massoniana plantations in subtropical China. *Forest Ecology and Management, 495*. doi:10.1016/j.foreco.2021.119358

Jiang, Y., Lei, Y., Qin, W., Korpelainen, H., & Li, C. (2019). Revealing microbial processes and nutrient limitation in soil through ecoenzymatic stoichiometry and glomalin-related soil proteins in a retreating glacier forefield. *Geoderma, 338*, 313-324. doi:10.1016/j.geoderma.2018.12.023

Jin, V. L., & Evans, R. D. (2006). Elevated CO_2_ increases microbial carbon substrate use and nitrogen cycling in Mojave Desert soils. *Global Change Biology, 13*(2), 452-465. doi:10.1111/j.1365-2486.2006.01308.x

Jing, X., Chen, X., Tang, M., Ding, Z., Jiang, L., Li, P., . . . Zhu, B. (2017). Nitrogen deposition has minor effect on soil extracellular enzyme activities in six Chinese forests. *Science of the Total Environment, 607-608*, 806-815. doi:10.1016/j.scitotenv.2017.07.060

Jing, X., Chen, X., Xiao, W., Lin, L., Wang, C., He, J.-S., & Zhu, B. (2018). Soil enzymatic responses to multiple environmental drivers in the Tibetan grasslands: Insights from two manipulative field experiments and a meta-analysis. *Pedobiologia, 71*, 50-58. doi:10.1016/j.pedobi.2018.10.001

Kandeler, E., Gebala, A., Boeddinghaus, R. S., Müller, K., Rennert, T., Soares, M., . . . Marhan, S. (2019). The mineralosphere – Succession and physiology of bacteria and fungi colonising pristine minerals in grassland soils under different land-use intensities. *Soil Biology and Biochemistry, 136*. doi:10.1016/j.soilbio.2019.107534

Kardol, P., Cregger, M. A., Campany, C. E., & Classen, A. T. (2010). Soil ecosystem functioning under climate change: plant species and community effects. *Ecology, 91*(3), 767-781. doi:10.1890/09-0135.1

Kaštovská, E., Straková, P., Edwards, K., Urbanová, Z., Bárta, J., Mastný, J., . . . Picek, T. (2017). Cotton-Grass and Blueberry have Opposite Effect on Peat Characteristics and Nutrient Transformation in Peatland. *Ecosystems, 21*(3), 443-458. doi:10.1007/s10021-017-0159-3

Keeler, B. L., Hobbie, S. E., & Kellogg, L. E. (2008). Effects of long-term nitrogen addition on microbial enzyme activity in eight forested and grassland sites: implications for litter and soil organic matter decomposition. *Ecosystems, 12*(1), 1-15. doi:10.1007/s10021-008-9199-z

Khomutova, T. E., Fornasier, F., Yeltsov, M. V., Chernysheva, E. V., & Borisov, A. V. (2021). Influence of grazing on the structure and biological activity of dry steppe soils of the southern Russian Plain. *Land Degradation & Development, 32*(17), 4832-4844. doi:10.1002/ldr.4032

King, A. J., Meyer, A. F., & Schmidt, S. K. (2008). High levels of microbial biomass and activity in unvegetated tropical and temperate alpine soils. *Soil Biology and Biochemistry, 40*(10), 2605-2610. doi:10.1016/j.soilbio.2008.06.026

Koyama, A., Steinweg, J. M., Haddix, M. L., Dukes, J. S., & Wallenstein, M. D. (2018). Soil bacterial community responses to altered precipitation and temperature regimes in an old field grassland are mediated by plants. *FEMS Microbiology Ecology, 94*(1). doi:10.1093/femsec/fix156

Kunito, T., Isomura, I., Sumi, H., Park, H.-D., Toda, H., Otsuka, S., . . . Senoo, K. (2016). Aluminum and acidity suppress microbial activity and biomass in acidic forest soils. *Soil Biology and Biochemistry, 97*, 23-30. doi:10.1016/j.soilbio.2016.02.019

Li, G., Kim, S., Han, S. H., Chang, H., Du, D. L., & Son, Y. (2018). Precipitation affects soil microbial and extracellular enzymatic responses to warming. *Soil Biology & Biochemistry, 120*, 212-221. doi:10.1016/j.soilbio.2018.02.014

Li, J., Nie, M., & Pendall, E. (2020). Soil physico-chemical properties are more important than microbial diversity and enzyme activity in controlling carbon and nitrogen stocks near Sydney, Australia. *Geoderma, 366*. doi:10.1016/j.geoderma.2020.114201

Li, Q., Liu, Y., Gu, Y., Guo, L., Huang, Y., Zhang, J., . . . Zhu, P. (2020). Ecoenzymatic stoichiometry and microbial nutrient limitations in rhizosphere soil along the Hailuogou Glacier forefield chronosequence. *Science of the Total Environment, 704*, 135413. doi:10.1016/j.scitotenv.2019.135413

Li, T., Bu, Z., Liu, W., Zhang, M., Peng, C., Zhu, Q., . . . Wang, M. (2019). Weakening of the ‘enzymatic latch’ mechanism following long-term fertilization in a minerotrophic peatland. *Soil Biology and Biochemistry, 136*. doi:10.1016/j.soilbio.2019.107528

Li, T., Ge, L., Huang, J., Yuan, X., Peng, C., Wang, S., . . . Wang, M. (2020). Contrasting responses of soil exoenzymatic interactions and the dissociated carbon transformation to short- and long-term drainage in a minerotrophic peatland. *Geoderma, 377*. doi:10.1016/j.geoderma.2020.114585

Li, T., Wang, R., Cai, J., Meng, Y., Wang, Z., Feng, X., Liu, H., Turco, R.F. and Jiang, Y. (2021). Enhanced carbon acquisition and use efficiency alleviate microbial carbon relative to nitrogen limitation under soil acidification. Ecological Processes, 10, 32. doi:10.1186/s13717-021-00309-1

Li, W., Chen, X., Liu, M., Kuzyakov, Y., Jiang, C., Wu, M., & Li, Z. (2017). Shifts in microbial communities with increasing soil fertility across a chronosequence of paddy cultivation in subtropical China. *Applied Soil Ecology, 120*, 153-159. doi:10.1016/j.apsoil.2017.07.031

Li, Y., Qing, Y., Lyu, M., Chen, S., Yang, Z., Lin, C., & Yang, Y. (2018). Effects of artificial warming on different soil organic carbon and nitrogen pools in a subtropical plantation. *Soil Biology and Biochemistry, 124*, 161-167. doi:10.1016/j.soilbio.2018.06.007

Li, Y., Zhou, H., Chen, W., Wu, Y., Qiao, L., Yan, Z., . . . Xue, S. (2021). Long-term warming does not affect soil ecoenzyme activity and original microbial nutrient limitation on the Qinghai—Tibet Plateau. *Soil Ecology Letters, 4*(4), 383-398. doi:10.1007/s42832-021-0116-0

Liao, H., Sheng, M., Liu, J., Ai, X., Li, C., Ai, S., & Ai, Y. (2021). Soil N availability drives the shifts of enzyme activity and microbial phosphorus limitation in the artificial soil on cut slope in southwestern China. *Environmental Science and Pollution Research,* 28:33307–33319. doi:10.1007/s11356-021-13012-7

Liu, J., Chen, J., Chen, G., Guo, J., & Li, Y. (2020). Enzyme stoichiometry indicates the variation of microbial nutrient requirements at different soil depths in subtropical forests. *PLoS One, 15*(2), e0220599. doi:10.1371/journal.pone.0220599

Liu, R., Zhang, Y., Hu, X.-F., Wan, S., Wang, H., Liang, C., & Chen, F.-S. (2021). Litter manipulation effects on microbial communities and enzymatic activities vary with soil depth in a subtropical Chinese fir plantation. *Forest Ecology and Management, 480*. doi:10.1016/j.foreco.2020.118641

Liu, Y., Chen, Q., Wang, Z., Zheng, H., Chen, Y., Chen, X., . . . Zhang, J. (2019). Nitrogen addition alleviates microbial nitrogen limitations and promotes soil respiration in a subalpine coniferous forest. *Forests, 10*(11). doi:10.3390/f10111038

Liu, Y., Shen, X., Chen, Y. M., Wang, L. F., Chen, Q. M., Zhang, J., . . . Chen, L. H. (2019). Litter chemical quality strongly affects forest floor microbial groups and ecoenzymatic stoichiometry in the subalpine forest. *Annals of Forest Science, 76*(4). doi:10.1007/s13595-019-0890-3

Loeppmann, S., Blagodatskaya, E., Pausch, J., & Kuzyakov, Y. (2016). Enzyme properties down the soil profile - A matter of substrate quality in rhizosphere and detritusphere. *Soil Biology and Biochemistry, 103*, 274-283. doi:10.1016/j.soilbio.2016.08.023

Loeppmann, S., Breidenbach, A., Spielvogel, S., Dippold, M. A., & Blagodatskaya, E. (2020). Organic nutrients induced coupled C- and P-cycling enzyme activities during microbial growth in forest soils. *Frontiers in Forests and Global Change, 3*. doi:10.3389/ffgc.2020.00100

Luan, L., Liu, E., Gu, X., & Sun, J. (2020). Effects of litter manipulation and nitrogen addition on soil ecoenzymatic stoichiometry in a mixed pine and oak forest. *Acta Ecologica Sinica, 40*(24), 9220-9233. doi:10.5846/stxb202001030024

Luo, G., Ling, N., Xue, C., Dippold, M. A., Firbank, L. G., Guo, S., . . . Shen, Q. (2019). Nitrogen-inputs regulate microbial functional and genetic resistance and resilience to drying–rewetting cycles, with implications for crop yields. *Plant and Soil, 441*(1-2), 301-315. doi:10.1007/s11104-019-04120-y

Luo, G., Rensing, C., Chen, H., Liu, M., Wang, M., Guo, S., . . . Briones, M. (2018). Deciphering the associations between soil microbial diversity and ecosystem multifunctionality driven by long‐term fertilization management. *Functional Ecology, 32*(4), 1103-1116. doi:10.1111/1365-2435.13039

Luo, L., Zhu, L., Hong, W., Gu, J.-D., Shi, D., He, Y., . . . Deng, O. (2021). Microbial resource limitation and regulation of soil carbon cycle in Zoige Plateau peatland soils. *Catena, 205*. doi:10.1016/j.catena.2021.105478

Ma, S., Chen, G., Tian, D., Du, E., Xiao, W., Jiang, L., . . . Fang, J. (2020). Effects of seven-year nitrogen and phosphorus additions on soil microbial community structures and residues in a tropical forest in Hainan Island, China. *Geoderma, 361*. doi:10.1016/j.geoderma.2019.114034

Ma, Z., Zhang, X., Zheng, B., Yue, S., Zhang, X., Zhai, B., . . . Razavi, B. S. (2021). Effects of plastic and straw mulching on soil microbial P limitations in maize fields: Dependency on soil organic carbon demonstrated by ecoenzymatic stoichiometry. *Geoderma, 388*. doi:10.1016/j.geoderma.2021.114928

Mannisto, M., Vuosku, J., Stark, S., Saravesi, K., Suokas, M., Markkola, A., . . . Rautio, P. (2018). Bacterial and fungal communities in boreal forest soil are insensitive to changes in snow cover conditions. *FEMS Microbiology Ecology, 94*(9). doi:10.1093/femsec/fiy123

Mganga, K. Z., Razavi, B. S., & Kuzyakov, Y. (2015). Microbial and enzymes response to nutrient additions in soils of Mt. Kilimanjaro region depending on land use. *European Journal of Soil Biology, 69*, 33-40. doi:10.1016/j.ejsobi.2015.05.001

Mganga, K. Z., Razavi, B. S., & Kuzyakov, Y. (2016). Land use affects soil biochemical properties in Mt. Kilimanjaro region. *Catena, 141*, 22-29. doi:10.1016/j.catena.2016.02.013

Min, K., Buckeridge, K., Ziegler, S. E., Edwards, K. A., Bagchi, S., & Billings, S. A. (2019). Temperature sensitivity of biomass-specific microbial exo-enzyme activities and CO_2_ efflux is resistant to change across short- and long-term timescales. *Global Change Biology, 25*(5), 1793-1807. doi:10.1111/gcb.14605

Miura, M., Jones, T. G., Hill, P. W., & Jones, D. L. (2019). Freeze-thaw and dry-wet events reduce microbial extracellular enzyme activity, but not organic matter turnover in an agricultural grassland soil. *Applied Soil Ecology, 144*, 196-199. doi:10.1016/j.apsoil.2019.08.002

Moorhead, D. L., Rinkes, Z. L., Sinsabaugh, R. L., & Weintraub, M. N. (2013). Dynamic relationships between microbial biomass, respiration, inorganic nutrients and enzyme activities: informing enzyme-based decomposition models. *Frontiers in Microbiology, 4*, 223. doi:10.3389/fmicb.2013.00223

Mori, T., Wang, S. H., Zhang, W., & Mo, J. M. (2019). A potential source of soil ecoenzymes: From the phyllosphere to soil via throughfall. *Applied Soil Ecology, 139*, 25-28. doi:10.1016/j.apsoil.2019.02.004

Moscatelli, M. C., Lagomarsino, A., Angelis, P. D., & Grego, S. (2005). Seasonality of soil biological properties in a poplar plantation growing under elevated atmospheric CO_2_. *Applied Soil Ecology, 30*(3), 162-173. doi:10.1016/j.apsoil.2005.02.008

Nemergut, D. R., Townsend, A. R., Sattin, S. R., Freeman, K. R., Fierer, N., Neff, J. C., . . . Schmidt, S. K. (2008). The effects of chronic nitrogen fertilization on alpine tundra soil microbial communities: implications for carbon and nitrogen cycling. *Environmental Microbiology, 10*(11), 3093-3105. doi:10.1111/j.1462-2920.2008.01735.x

Nisson, D. M., & Allison, S. D. (2020). Litter microbial respiration and enzymatic resistance to drought stress. *Elementa-Science of the Anthropocene, 8*. doi:10.1525/elementa.442

Nottingham, A. T., Meir, P., Velasquez, E., & Turner, B. L. (2020). Soil carbon loss by experimental warming in a tropical forest. *Nature, 584*(7820), 234-237. doi:10.1038/s41586-020-2566-4

Nottingham, A. T., Turner, B. L., Chamberlain, P. M., Stott, A. W., & Tanner, E. V. J. (2011). Priming and microbial nutrient limitation in lowland tropical forest soils of contrasting fertility. *Biogeochemistry, 111*(1-3), 219-237. doi:10.1007/s10533-011-9637-4

Nottingham, A. T., Turner, B. L., Whitaker, J., Ostle, N., Bardgett, R. D., McNamara, N. P., . . . Meir, P. (2016). Temperature sensitivity of soil enzymes along an elevation gradient in the Peruvian Andes. *Biogeochemistry, 127*(2-3), 217-230. doi:10.1007/s10533-015-0176-2

Nottingham, A. T., Turner, B. L., Whitaker, J., Ostle, N. J., McNamara, N. P., Bardgett, R. D., . . . Meir, P. (2015). Soil microbial nutrient constraints along a tropical forest elevation gradient: a belowground test of a biogeochemical paradigm. *Biogeosciences, 12*(20), 6071-6083. doi:10.5194/bg-12-6071-2015

Nottingham, A. T., Whitaker, J., Ostle, N. J., Bardgett, R. D., McNamara, N. P., Fierer, N., . . . Meir, P. (2019). Microbial responses to warming enhance soil carbon loss following translocation across a tropical forest elevation gradient. *Ecology Letters, 22*(11), 1889-1899. doi:10.1111/ele.13379

Ochoa-Hueso, R., Borer, E. T., Seabloom, E. W., Hobbie, S. E., Risch, A. C., Collins, S. L., . . . Zamin, T. (2020). Microbial processing of plant remains is co-limited by multiple nutrients in global grasslands. *Global Change Biology, 26*(8), 4572-4582. doi:10.1111/gcb.15146

Peng, X., & Wang, W. (2016). Stoichiometry of soil extracellular enzyme activity along a climatic transect in temperate grasslands of northern China. *Soil Biology and Biochemistry, 98*, 74-84. doi:10.1016/j.soilbio.2016.04.008

Perez-Mon, C., Frey, B., & Frossard, A. (2020). Functional and structural responses of arctic and alpine soil prokaryotic and fungal communities under freeze-thaw cycles of different frequencies. *Frontiers in Microbiology, 11*, 982. doi:10.3389/fmicb.2020.00982

Phillips, L. A., Ward, V., & Jones, M. D. (2014). Ectomycorrhizal fungi contribute to soil organic matter cycling in sub-boreal forests. *The ISME Journal, 8*(3), 699-713. doi:10.1038/ismej.2013.195

Pinsonneault, A. J., Moore, T. R., & Roulet, N. T. (2016). Effects of long-term fertilization on peat stoichiometry and associated microbial enzyme activity in an ombrotrophic bog. *Biogeochemistry, 129*(1-2), 149-164. doi:10.1007/s10533-016-0224-6

Piton, G., Legay, N., Arnoldi, C., Lavorel, S., Clément, J.-C., Foulquier, A., & Lamb, E. (2020). Using proxies of microbial community‐weighted means traits to explain the cascading effect of management intensity, soil and plant traits on ecosystem resilience in mountain grasslands. *Journal of Ecology, 108*(3), 876-893. doi:10.1111/1365-2745.13327

Preston, M. D., Smemo, K. A., McLaughlin, J. W., & Basiliko, N. (2012). Peatland microbial communities and decomposition processes in the james bay lowlands, Canada. *Frontiers in Microbiology, 3*, 70. doi:10.3389/fmicb.2012.00070

Qiang, W., He, L., Zhang, Y., Liu, B., Liu, Y., Liu, Q., & Pang, X. (2021). Aboveground vegetation and soil physicochemical properties jointly drive the shift of soil microbial community during subalpine secondary succession in southwest China. *Catena, 202*. doi:10.1016/j.catena.2021.105251

Qin, L., Freeman, C., Jia, X., Zhang, Z., Liu, B., Zhang, S., & Jiang, M. (2021). Microbial enzyme activity and stoichiometry signal the effects of agricultural intervention on nutrient cycling in peatlands. *Ecological Indicators, 122*. doi:10.1016/j.ecolind.2020.107242

Ramirez, K. S., Craine, J. M., & Fierer, N. (2012). Consistent effects of nitrogen amendments on soil microbial communities and processes across biomes. *Global Change Biology, 18*(6), 1918-1927. doi:10.1111/j.1365-2486.2012.02639.x

Riggs, C. E., & Hobbie, S. E. (2016). Mechanisms driving the soil organic matter decomposition response to nitrogen enrichment in grassland soils. *Soil Biology and Biochemistry, 99*, 54-65. doi:10.1016/j.soilbio.2016.04.023

Schnecker, J., Wild, B., Takriti, M., Eloy Alves, R. J., Gentsch, N., Gittel, A., . . . Richter, A. (2015). Microbial community composition shapes enzyme patterns in topsoil and subsoil horizons along a latitudinal transect in Western Siberia. *Soil Biology and Biochemistry, 83*, 106-115. doi:10.1016/j.soilbio.2015.01.016

Scola, V., Ramond, J. B., Frossard, A., Zablocki, O., Adriaenssens, E. M., Johnson, R. M., . . . Cowan, D. A. (2018). Namib desert soil microbial community diversity, assembly, and function along a natural xeric gradient. *Microbial Ecology, 75*(1), 193-203. doi:10.1007/s00248-017-1009-8

Shahariar, S., Helgason, B., Soolanayakanahally, R., & Bedard-Haughn, A. (2021). Soil enzyme activity as affected by land-use, salinity, and groundwater fluctuations in wetland soils of the prairie pothole region. *Wetlands, 41*(2). doi:10.1007/s13157-021-01431-8

Sheng-Ji, Y., Li, B.-Y., Gao, D.-X., Fu, S.-Y., Lu, Y.-F., Xu, M.-P., . . . Han, X.-H. (2020). Response of ecoenzymatic stoichiometry to soil physicochemical properties after afforestation on loess hilly region. *Eurasian Soil Science, 53*(11), 1669-1675. doi:10.1134/s1064229320110125

Shvaleva, A., Siljanen, H. M., Correia, A., Costa, E. S. F., Lamprecht, R. E., Lobo-do-Vale, R., . . . Martikainen, P. J. (2015). Environmental and microbial factors influencing methane and nitrous oxide fluxes in Mediterranean cork oak woodlands: trees make a difference. *Frontiers in Microbiology, 6*, 1104. doi:10.3389/fmicb.2015.01104

Sinsabaugh, R. L., Belnap, J., Rudgers, J., Kuske, C. R., Martinez, N., & Sandquist, D. (2015). Soil microbial responses to nitrogen addition in arid ecosystems. *Frontiers in Microbiology, 6*, 819. doi:10.3389/fmicb.2015.00819

Sinsabaugh, R. L., Lauber, C. L., Weintraub, M. N., Ahmed, B., Allison, S. D., Crenshaw, C., . . . Zeglin, L. H. (2008). Stoichiometry of soil enzyme activity at global scale. *Ecology Letters, 11*(11), 1252-1264. doi:10.1111/j.1461-0248.2008.01245.x

Smemo, K. A., Petersen, S. M., Kluber, L. A., Shaw, A. N., & DeForest, J. L. (2021). Temporal soil enzyme patterns provide new insights into the nutrient economy of acidic hardwood forests. *Biogeochemistry, 155*(1), 97-112. doi:10.1007/s10533-021-00814-7

Sorensen, P. O., Finzi, A. C., Giasson, M.-A., Reinmann, A. B., Sanders-DeMott, R., & Templer, P. H. (2018). Winter soil freeze-thaw cycles lead to reductions in soil microbial biomass and activity not compensated for by soil warming. *Soil Biology and Biochemistry, 116*, 39-47. doi:10.1016/j.soilbio.2017.09.026

Souza-Alonso, P., Novoa, A., & González, L. (2014). Soil biochemical alterations and microbial community responses under Acacia dealbata Link invasion. *Soil Biology and Biochemistry, 79*, 100-108. doi:10.1016/j.soilbio.2014.09.008

Stark, S., Egelkraut, D., Aronsson, K. Å., & Olofsson, J. (2019). Contrasting vegetation states do not diverge in soil organic matter storage: evidence from historical sites in tundra. *Ecology, 100*(7), e02731. doi:10.1002/ecy.2731

Stark, S., Ylänne, H., & Tolvanen, A. (2018). Long-term warming alters soil and enzymatic N:P stoichiometry in subarctic tundra. *Soil Biology and Biochemistry, 124*, 184-188. doi:10.1016/j.soilbio.2018.06.016

Stock, S. C., Köster, M., Dippold, M. A., Nájera, F., Matus, F., Merino, C., . . . Kuzyakov, Y. (2019). Environmental drivers and stoichiometric constraints on enzyme activities in soils from rhizosphere to continental scale. *Geoderma, 337*, 973-982. doi:10.1016/j.geoderma.2018.10.030

Stursova, M., Crenshaw, C. L., & Sinsabaugh, R. L. (2006). Microbial responses to long-term N deposition in a semiarid grassland. *Microbial Ecology, 51*(1), 90-98. doi:10.1007/s00248-005-5156-y

Suding, K. N., Ashton, I. W., Bechtold, H., Bowman, W. D., Mobley, M. L., & Winkleman, R. (2008). Plant and microbe contribution to community resilience in a directionally changing environment. *Ecological Monographs, 78*(3), 313-329. doi:10.1890/07-1092.1

Tapia-Torres, Y., Elser, J. J., Souza, V., & García-Oliva, F. (2015). Ecoenzymatic stoichiometry at the extremes: How microbes cope in an ultra-oligotrophic desert soil. *Soil Biology and Biochemistry, 87*, 34-42. doi:10.1016/j.soilbio.2015.04.007

Tatariw, C., MacRae, J. D., Fernandez, I. J., Gruselle, M.-C., Salvino, C. J., & Simon, K. S. (2017). Chronic nitrogen enrichment at the watershed scale does not enhance microbial phosphorus limitation. *Ecosystems, 21*(1), 178-189. doi:10.1007/s10021-017-0140-1

Tischer, A., Blagodatskaya, E., & Hamer, U. (2014). Extracellular enzyme activities in a tropical mountain rainforest region of southern Ecuador affected by low soil P status and land-use change. *Applied Soil Ecology, 74*, 1-11. doi:10.1016/j.apsoil.2013.09.007

Truong, C., Gabbarini, L. A., Corrales, A., Mujic, A. B., Escobar, J. M., Moretto, A., & Smith, M. E. (2019). Ectomycorrhizal fungi and soil enzymes exhibit contrasting patterns along elevation gradients in southern Patagonia. *New Phytologist, 222*(4), 1936-1950. doi:10.1111/nph.15714

Turner, B. L. (2010). Variation in pH optima of hydrolytic enzyme activities in tropical rain forest soils. *Applied and Environmental Microbiology, 76*(19), 6485-6493. doi:10.1128/AEM.00560-10

Turner, B. L., & Romero, T. E. (2010). Stability of hydrolytic enzyme activity and microbial phosphorus during storage of tropical rain forest soils. *Soil Biology and Biochemistry, 42*(3), 459-465. doi:10.1016/j.soilbio.2009.11.029

Ullah, S., Ai, C., Huang, S., Zhang, J., Jia, L., Ma, J., . . . He, P. (2019). The responses of extracellular enzyme activities and microbial community composition under nitrogen addition in an upland soil. *PLoS One, 14*(9), e0223026. doi:10.1371/journal.pone.0223026

Verchot, L., & Borelli, T. (2005). Application of -nitrophenol (NP) enzyme assays in degraded tropical soils. *Soil Biology and Biochemistry, 37*(4), 625-633. doi:10.1016/j.soilbio.2004.09.005

Wang, C., Lu, X., Mori, T., Mao, Q., Zhou, K., Zhou, G., . . . Mo, J. (2018). Responses of soil microbial community to continuous experimental nitrogen additions for 13 years in a nitrogen-rich tropical forest. *Soil Biology and Biochemistry, 121*, 103-112. doi:10.1016/j.soilbio.2018.03.009

Wang, C., Ning, P., Li, J., Wei, X., Ge, T., Cui, Y., . . . Shen, W. (2022). Responses of soil microbial community composition and enzyme activities to long-term organic amendments in a continuous tobacco cropping system. *Applied Soil Ecology, 169*. doi:10.1016/j.apsoil.2021.104210

Wang, C., Zhang, R., Vilonen, L., Qu, Y., Fu, X., Shi, B., . . . Sun, W. (2021). Grazing and nitrogen addition restructure the spatial heterogeneity of soil microbial community structure and enzymatic activities. *Functional Ecology, 35*(12), 2763-2777. doi:10.1111/1365-2435.13926

Wang, J., Hayes, F., Turner, R., Chadwick, D. R., Mills, G., & Jones, D. L. (2019). Effects of four years of elevated ozone on microbial biomass and extracellular enzyme activities in a semi-natural grassland. *Science of the Total Environment, 660*, 260-268. doi:10.1016/j.scitotenv.2019.01.040

Wang, J., Wang, X., Liu, G., Wang, G., Wu, Y., & Zhang, C. (2020). Fencing as an effective approach for restoration of alpine meadows: Evidence from nutrient limitation of soil microbes. *Geoderma, 363*. doi:10.1016/j.geoderma.2019.114148

Wang, Q., Kwak, J.-H., Choi, W.-J., & Chang, S. X. (2018). Decomposition of trembling aspen leaf litter under long-term nitrogen and sulfur deposition: Effects of litter chemistry and forest floor microbial properties. *Forest Ecology and Management, 412*, 53-61. doi:10.1016/j.foreco.2018.01.042

Wang, R., Cao, Y., Wang, H., Dijkstra, F. A., Jiang, J., Zhao, R., . . . Peñuelas, J. (2019). Exogenous P compounds differentially interacted with N availability to regulate enzymatic activities in a meadow steppe. *European Journal of Soil Science, 71*(4), 667-680. doi:10.1111/ejss.12906

Wang, R., Dorodnikov, M., Yang, S., Zhang, Y., Filley, T. R., Turco, R. F., . . . Jiang, Y. (2015). Responses of enzymatic activities within soil aggregates to 9-year nitrogen and water addition in a semi-arid grassland. *Soil Biology and Biochemistry, 81*, 159-167. doi:10.1016/j.soilbio.2014.11.015

Wang, X., Cui, Y., Zhang, X., Ju, W., Duan, C., Wang, Y., & Fang, L. (2020). A novel extracellular enzyme stoichiometry method to evaluate soil heavy metal contamination: Evidence derived from microbial metabolic limitation. *Science of the Total Environment, 738*, 139709. doi:10.1016/j.scitotenv.2020.139709

Wang, Y., Shahbaz, M., Zhran, M., Chen, A., Zhu, Z., Galal, Y. G. M., . . . Li, Y. (2021). Microbial resource limitation in aggregates in karst and non-karst soils. *Agronomy, 11*(8). doi:10.3390/agronomy11081591

Waring, B. G., De Guzman, M. E., Du, D. V., Dupuy, J. M., Gei, M., Gutknecht, J., . . . Powers, J. S. (2021). Soil biogeochemistry across Central and South American tropical dry forests. *Ecological Monographs, 91*(3). doi:10.1002/ecm.1453

Warnock, D. D., Litvak, M. E., Morillas, L., & Sinsabaugh, R. L. (2016). Drought-induced piñon mortality alters the seasonal dynamics of microbial activity in piñon–juniper woodland. *Soil Biology and Biochemistry, 92*, 91-101. doi:10.1016/j.soilbio.2015.09.007

Weand, M. P., Arthur, M. A., Lovett, G. M., McCulley, R. L., & Weathers, K. C. (2010). Effects of tree species and N additions on forest floor microbial communities and extracellular enzyme activities. *Soil Biology and Biochemistry, 42*(12), 2161-2173. doi:10.1016/j.soilbio.2010.08.012

Weintraub, S. R., Wieder, W. R., Cleveland, C. C., & Townsend, A. R. (2012). Organic matter inputs shift soil enzyme activity and allocation patterns in a wet tropical forest. *Biogeochemistry, 114*(1-3), 313-326. doi:10.1007/s10533-012-9812-2

Wick, B., Tiessen, H., & Menezes, R. S. C. (2000). Land quality changes following the conversion of the natural vegetation into silvo-pastoral systems in semi-arid NE Brazil. *Plant and Soil, 222*(1-2), 59-70. doi:10.1023/A:1004756416281

Wu, Y., Chen, W., Li, Q., Guo, Z., Li, Y., Zhao, Z., . . . Xue, S. (2020). Ecoenzymatic stoichiometry and nutrient limitation under a natural secondary succession of vegetation on the Loess Plateau, China. *Land Degradation & Development, 32*(1), 399-409. doi:10.1002/ldr.3723

Xiao, H., Yang, H., Zhao, M., Monaco, T. A., Rong, Y., Huang, D., . . . Wang, D. (2021). Soil extracellular enzyme activities and the abundance of nitrogen-cycling functional genes responded more to N addition than P addition in an Inner Mongolian meadow steppe. *Science of the Total Environment, 759*, 143541. doi:10.1016/j.scitotenv.2020.143541

Xiao, L., Liu, G., Li, P., Li, Q., & Xue, S. (2020). Ecoenzymatic stoichiometry and microbial nutrient limitation during secondary succession of natural grassland on the Loess Plateau, China. *Soil and Tillage Research, 200*. doi:10.1016/j.still.2020.104605

Xu, H., Zhu, B., Wei, X., Yu, M., & Cheng, X. (2021). Root functional traits mediate rhizosphere soil carbon stability in a subtropical forest. *Soil Biology and Biochemistry, 162*. doi:10.1016/j.soilbio.2021.108431

Xu, Z., Yu, G., Zhang, X., He, N., Wang, Q., Wang, S., . . . Wang, C. (2017). Soil enzyme activity and stoichiometry in forest ecosystems along the North-South Transect in eastern China (NSTEC). *Soil Biology and Biochemistry, 104*, 152-163. doi:10.1016/j.soilbio.2016.10.020

Yan, B., Sun, Y., He, G., He, R., Zhang, M., Fang, H., & Shi, L. (2020). Nitrogen enrichment affects soil enzymatic stoichiometry via soil acidification in arid and hot land. *Pedobiologia, 81-82*. doi:10.1016/j.pedobi.2020.150663

Yan, Z., Li, Y., Wu, H., Zhang, K., Hao, Y., Wang, J., . . . Kang, X. (2020). Different responses of soil hydrolases and oxidases to extreme drought in an alpine peatland on the Qinghai-Tibet Plateau, China. *European Journal of Soil Biology, 99*. doi:10.1016/j.ejsobi.2020.103195

Yang, Y., Liang, C., Wang, Y., Cheng, H., An, S., & Chang, S. X. (2020). Soil extracellular enzyme stoichiometry reflects the shift from P- to N-limitation of microorganisms with grassland restoration. *Soil Biology and Biochemistry, 149*. doi:10.1016/j.soilbio.2020.107928

Yao, Q., Li, Z., Song, Y., Wright, S.J., Guo, X., Tringe, S.G., Tfaily, M.M., Paša-Tolić, L., Hazen, T.C., Turner, B.L. and Mayes, M.A. (2018). Community proteogenomics reveals the systemic impact of phosphorus availability on microbial functions in tropical soil. Nature ecology & evolution, 2, 499-509. doi:10.1038/s41559-017-0463-5

Yao, X., Zeng, W., Zeng, H., & Wang, W. (2020). Soil microbial attributes along a chronosequence of Scots pine (Pinus sylvestris var. mongolica) plantations in northern China. *Pedosphere, 30*(4), 433-442. doi:10.1016/s1002-0160(17)60329-1

Yi, J., Zeng, Q., Mei, T., Zhang, S., Li, Q., Wang, M., & Tan, W. (2022). Disentangling drivers of soil microbial nutrient limitation in intensive agricultural and natural ecosystems. *Science of the Total Environment, 806*(Pt 1), 150555. doi:10.1016/j.scitotenv.2021.150555

Ylänne, H., Kaarlejärvi, E., Väisänen, M., Männistö, M. K., Ahonen, S. H. K., Olofsson, J., & Stark, S. (2020). Removal of grazers alters the response of tundra soil carbon to warming and enhanced nitrogen availability. *Ecological Monographs, 90*(1). doi:10.1002/ecm.1396

You, Y., Xu, H., Wu, X., Zhou, X., Tan, X., Li, M., . . . Huang, X. (2020). Native broadleaf tree species stimulate topsoil nutrient transformation by changing microbial community composition and physiological function, but not biomass in subtropical plantations with low P status. *Forest Ecology and Management, 477*. doi:10.1016/j.foreco.2020.118491

Yuan, X. B., Niu, D. C., Gherardi, L. A., Liu, Y. B., Wang, Y., Elser, J. J., & Fu, H. (2019). Linkages of stoichiometric imbalances to soil microbial respiration with increasing nitrogen addition: Evidence from a long-term grassland experiment. *Soil Biology & Biochemistry, 138*. doi:10.1016/j.soilbio.2019.107580

Yuan, Y., Li, Y., Mou, Z., Kuang, L., Wu, W., Zhang, J., . . . Liu, Z. (2021). Phosphorus addition decreases microbial residual contribution to soil organic carbon pool in a tropical coastal forest. *Global Change Biology, 27*(2), 454-466. doi:10.1111/gcb.15407

Zeglin, L. H., Stursova, M., Sinsabaugh, R. L., & Collins, S. L. (2007). Microbial responses to nitrogen addition in three contrasting grassland ecosystems. *Oecologia, 154*(2), 349-359. doi:10.1007/s00442-007-0836-6

Zhang, C., Li, J., Wang, J., Liu, G., Wang, G., Guo, L., & Peng, S. (2019). Decreased temporary turnover of bacterial communities along soil depth gradient during a 35-year grazing exclusion period in a semiarid grassland. *Geoderma, 351*, 49-58. doi:10.1016/j.geoderma.2019.05.010

Zhang, J., Ai, Z., Liang, C., Wang, G., Liu, G., & Xue, S. (2019). How microbes cope with short-term N addition in a Pinus tabuliformis forest-ecological stoichiometry. *Geoderma, 337*, 630-640. doi:10.1016/j.geoderma.2018.10.017

Zhang, J., Yang, X., Song, Y., Liu, H., Wang, G., Xue, S., . . . Geissen, V. (2020). Revealing the nutrient limitation and cycling for microbes under forest management practices in the Loess Plateau – Ecological stoichiometry. *Geoderma, 361*. doi:10.1016/j.geoderma.2019.114108

Zhang, Q., Zhang, D., Wu, J., Li, J., Feng, J., & Cheng, X. (2021). Soil nitrogen‐hydrolyzing enzyme activity and stoichiometry following a subtropical land use change. *Land Degradation & Development, 32*(15), 4277-4287. doi:10.1002/ldr.4034

Zhang, W., Xu, Y., Gao, D., Wang, X., Liu, W., Deng, J., . . . Ren, G. (2019). Ecoenzymatic stoichiometry and nutrient dynamics along a revegetation chronosequence in the soils of abandoned land and Robinia pseudoacacia plantation on the Loess Plateau, China. *Soil Biology and Biochemistry, 134*, 1-14. doi:10.1016/j.soilbio.2019.03.017

Zhang, X., Dong, W., Dai, X., Schaeffer, S., Yang, F., Radosevich, M., . . . Sun, X. (2015). Responses of absolute and specific soil enzyme activities to long term additions of organic and mineral fertilizer. *Science of the Total Environment, 536*, 59-67. doi:10.1016/j.scitotenv.2015.07.043

Zhang, Y., Sun, C., Chen, Z., Zhang, G., Chen, L., & Wu, Z. (2019). Stoichiometric analyses of soil nutrients and enzymes in a Cambisol soil treated with inorganic fertilizers or manures for 26 years. *Geoderma, 353*, 382-390. doi:10.1016/j.geoderma.2019.06.026

Zhao, F. Z., Ren, C. J., Han, X. H., Yang, G. H., Wang, J., & Doughty, R. (2018). Changes of soil microbial and enzyme activities are linked to soil C, N and P stoichiometry in afforested ecosystems. *Forest Ecology and Management, 427*, 289-295. doi:10.1016/j.foreco.2018.06.011

Zheng, H., Liu, Y., Chen, Y., Zhang, J., Li, H., Wang, L., & Chen, Q. (2020). Short-term warming shifts microbial nutrient limitation without changing the bacterial community structure in an alpine timberline of the eastern Tibetan Plateau. *Geoderma, 360*. doi:10.1016/j.geoderma.2019.113985

Zheng, L., Chen, H., Wang, Y., Mao, Q., Zheng, M., Su, Y., . . . Li, D. (2020). Responses of soil microbial resource limitation to multiple fertilization strategies. *Soil and Tillage Research, 196*. doi:10.1016/j.still.2019.104474

Zheng, W., Zhao, Z., Gong, Q., Zhai, B., & Li, Z. (2018). Responses of fungal–bacterial community and network to organic inputs vary among different spatial habitats in soil. *Soil Biology and Biochemistry, 125*, 54-63. doi:10.1016/j.soilbio.2018.06.029

Zheng, W., Zhao, Z. Y., Lv, F. L., Wang, R., Gong, Q. L., Zhai, B. N., . . . Li, Z. Y. (2019). Metagenomic exploration of the interactions between N and P cycling and SOM turnover in an apple orchard with a cover crop fertilized for 9 years. *Biology and Fertility of Soils, 55*(4), 365-381. doi:10.1007/s00374-019-01356-9

Zhong, Z., Li, W., Lu, X., Gu, Y., Wu, S., Shen, Z., . . . Ren, C. (2020). Adaptive pathways of soil microorganisms to stoichiometric imbalances regulate microbial respiration following afforestation in the Loess Plateau, China. *Soil Biology and Biochemistry, 151*. doi:10.1016/j.soilbio.2020.108048

Zhou, L., Liu, S., Shen, H., Zhao, M., Xu, L., Xing, A., . . . Sayer, E. (2020). Soil extracellular enzyme activity and stoichiometry in China's forests. *Functional Ecology, 34*(7), 1461-1471. doi:10.1111/1365-2435.13555

Zhou, X., Chen, C., Wang, Y., Xu, Z., Han, H., Li, L., & Wan, S. (2013). Warming and increased precipitation have differential effects on soil extracellular enzyme activities in a temperate grassland. *Science of the Total Environment, 444*, 552-558. doi:10.1016/j.scitotenv.2012.12.023

Zhou, Z., Zhang, H., Yuan, Z., & Gong, R. (2019). The nutrient release rate accounts for the effect of organic matter type on soil microbial carbon use efficiency of a Pinus tabulaeformis forest in northern China. *Journal of Soils and Sediments, 20*(1), 352-364. doi:10.1007/s11368-019-02423-2

Zhu, X., Liu, M., Kou, Y., Liu, D., Liu, Q., Zhang, Z., . . . Yin, H. (2020). Differential effects of N addition on the stoichiometry of microbes and extracellular enzymes in the rhizosphere and bulk soils of an alpine shrubland. *Plant and Soil, 449*(1-2), 285-301. doi:10.1007/s11104-020-04468-6

Zuccarini, P., Asensio, D., Ogaya, R., Sardans, J., & Penuelas, J. (2020). Effects of seasonal and decadal warming on soil enzymatic activity in a P-deficient Mediterranean shrubland. *Global Change Biology, 26*(6), 3698-3714. doi:10.1111/gcb.15077

Zuo, Y., Li, J., Zeng, H., & Wang, W. (2018). Vertical pattern and its driving factors in soil extracellular enzyme activity and stoichiometry along mountain grassland belts. *Biogeochemistry, 141*(1), 23-39. doi:10.1007/s10533-018-0499-x
