# Supplementary material for: Global patterns and drivers of soil microbial nitrogen and phosphorus use efficiency
Source: Nat Commun. 2026 Mar 17;17:2576. doi: 10.1038/s41467-026-70602-0 (PMC12999993; doi:10.1038/s41467-026-70602-0)
Supplement: Supplementary file 4 — Reporting Summary [file 41467_2026_70602_MOESM4_ESM.pdf]

nature portfolio

Corresponding author(s): Yongxing Cui

Last updated by author(s): Feb 4, 2026

Reporting Summary

Nature Portfolio wishes to improve the reproducibility of the work that we publish. This form provides structure for consistency and transparency in reporting. For further information on Nature Portfolio policies, see our Editorial Policies and the Editorial Policy Checklist.

Statistics

For all statistical analyses, confirm that the following items are present in the figure legend, table legend, main text, or Methods section.

n/a	Confirmed	
☐	☒	The exact sample size (n) for each experimental group/condition, given as a discrete number and unit of measurement
☐	☒	A statement on whether measurements were taken from distinct samples or whether the same sample was measured repeatedly
☐	☒	The statistical test(s) used AND whether they are one- or two-sided<br>*Only common tests should be described solely by name; describe more complex techniques in the Methods section.*
☒	☐	A description of all covariates tested
☐	☒	A description of any assumptions or corrections, such as tests of normality and adjustment for multiple comparisons
☐	☒	A full description of the statistical parameters including central tendency (e.g. means) or other basic estimates (e.g. regression coefficient) AND variation (e.g. standard deviation) or associated estimates of uncertainty (e.g. confidence intervals)
☐	☒	For null hypothesis testing, the test statistic (e.g. F, t, r) with confidence intervals, effect sizes, degrees of freedom and P value noted<br>*Give P values as exact values whenever suitable.*
☒	☐	For Bayesian analysis, information on the choice of priors and Markov chain Monte Carlo settings
☒	☐	For hierarchical and complex designs, identification of the appropriate level for tests and full reporting of outcomes
☒	☐	Estimates of effect sizes (e.g. Cohen's d, Pearson's r), indicating how they were calculated

Our web collection on statistics for biologists contains articles on many of the points above.

Software and code

Policy information about availability of computer code

Data collection	Numerical data extracted from figures were obtained using GetData Graph Digitizer software (version 2.26, http://www.getdata-graph-digitizer.com/index.php).<br>Soil pH, SOC, TN, and TP were obtained from the SoilGrids database (https://soilgrids.org/), while MAT and MAP were derived from WorldClim (https://www.worldclim.org/).
Data analysis	All statistical analyses and visualizations were performed using ArcGIS 10.5 and the R environment (v. 4.0.2)

For manuscripts utilizing custom algorithms or software that are central to the research but not yet described in published literature, software must be made available to editors and reviewers. We strongly encourage code deposition in a community repository (e.g. GitHub). See the Nature Portfolio guidelines for submitting code & software for further information.

Data

Policy information about availability of data

All manuscripts must include a data availability statement. This statement should provide the following information, where applicable:

- Accession codes, unique identifiers, or web links for publicly available datasets
- A description of any restrictions on data availability
- For clinical datasets or third party data, please ensure that the statement adheres to our policy

The data and R code in this study are publicly available at https://doi.org/10.6084/m9.figshare.30391051.

Research involving human participants, their data, or biological material

Policy information about studies with human participants or human data. See also policy information about sex, gender (identity/presentation), and sexual orientation and race, ethnicity and racism.

Reporting on sex and gender	N/A
Reporting on race, ethnicity, or other socially relevant groupings	N/A
Population characteristics	N/A
Recruitment	N/A
Ethics oversight	N/A

Note that full information on the approval of the study protocol must also be provided in the manuscript.

Field-specific reporting

Please select the one below that is the best fit for your research. If you are not sure, read the appropriate sections before making your selection.

☐ Life sciences ☐ Behavioural & social sciences ☒ Ecological, evolutionary & environmental sciences

For a reference copy of the document with all sections, see nature.com/documents/nr-reporting-summary-flat.pdf

Ecological, evolutionary & environmental sciences study design

All studies must disclose on these points even when the disclosure is negative.

Study description	This study provides the first global assessment of microbial nitrogen (NUE) and phosphorus use efficiencies (PUE) using an ecoenzymatic stoichiometric approach. We compiled data from 265 published studies (2,012 NUE, 3,419 PUE observations) and applied stoichiometric models combined with Random Forest upscaling to 1 km resolution. Key findings include: global average NUE (0.60) is nearly twice PUE (0.35), soil organic carbon (SOC) is the strongest predictor, and spatial patterns show notably lower NUE in tundra/boreal soils, while P limitation appears widespread. These results offer crucial parameters for large-scale soil carbon and nutrient cycle models.
Research sample	This study utilized a global dataset of microbial nitrogen (NUE) and phosphorus use efficiencies (PUE) derived from 265 published studies representing diverse terrestrial ecosystems. Paired measurements (NUE: n=2,012; PUE: n=3,149) were extracted from 12 distinct biomes (boreal forest, temperate forest, tropical forest, grassland, tundra, wetland, cropland) across the globe. Data were sourced from Web of Science and Google Scholar using specific keywords related to extracellular enzyme activities (β-glucosaminidase, β-glucosidase, leucine-amino-peptidase, acid phosphatase) measured via fluorogenic substrates. The sample represents microbial communities in natural and minimally manipulated ecosystems (control plots of manipulative experiments), intended to capture broad patterns of nutrient use efficiency across terrestrial biomes.
Sampling strategy	This study employed a global sampling strategy, extracting data from 265 peer-reviewed publications published up to 2022. Data were sourced from Web of Science and Google Scholar using keywords related to extracellular enzyme activities (β-glucosidase, N-acetyl-glucosaminidase, leucine-amino-peptidase, acid phosphatase). Studies were included if they provided data on all four enzyme activities from control plots in manipulative experiments, ensuring minimal experimental interference.
Data collection	Data collection involved compiling comprehensive datasets from selected studies, including:<br>Geographic coordinates (latitude and longitude)<br>Biome type (11 distinct terrestrial biomes)<br>Climate variables (mean annual temperature (MAT), mean annual precipitation (MAP))<br>Soil properties (soil depth, pH, soil organic carbon (SOC), total nitrogen (TN), total phosphorus (TP), texture, microbial biomass (C, N, P))
Timing and spatial scale	Data collection occurred between 1990 and 2022. The spatial scale encompassed global terrestrial ecosystems, with data points distributed across diverse biomes and climatic zones. Extrapolation was performed at a 1 km resolution using Random Forest models.
Data exclusions	Data exclusions were applied to ensure data quality and relevance:<br>Studies with altered nutrient inputs or other manipulations were excluded.<br>Outliers identified using the interquartile range (IQR) method were rigorously reviewed and excluded if erroneous.
Reproducibility	All analyses were conducted using established statistical methods and software (R, ArcGIS). The study design, data processing steps, and analytical methods are fully documented to ensure reproducibility.
Randomization	Randomization was not applicable to field sampling but was used in model selection (cross-validation) to ensure unbiased model performance assessment.
Blinding	Blinding was not applicable to this study, as data were collected from published literature.

Did the study involve field work? ☐ Yes ☒ No

Reporting for specific materials, systems and methods

We require information from authors about some types of materials, experimental systems and methods used in many studies. Here, indicate whether each material, system or method listed is relevant to your study. If you are not sure if a list item applies to your research, read the appropriate section before selecting a response.

Materials & experimental systems

n/a	Involved in the study
☒	☐ Antibodies
☒	☐ Eukaryotic cell lines
☒	☐ Palaeontology and archaeology
☒	☐ Animals and other organisms
☒	☐ Clinical data
☒	☐ Dual use research of concern
☒	☐ Plants

Methods

n/a	Involved in the study
☒	☐ ChIP-seq
☒	☐ Flow cytometry
☒	☐ MRI-based neuroimaging

Plants

Seed stocks	N/A
Novel plant genotypes	N/A
Authentication	N/A
